# Trajectory Matters: A Case of Bullet Extraction From the Heart via a Median Sternotomy

**DOI:** 10.7759/cureus.97820

**Published:** 2025-11-26

**Authors:** Omar G Bayyati, Hamad Al Habib, Bakir Bakir, Shirin H Alokayli

**Affiliations:** 1 Adult Cardiac Surgery, King Saud Medical City, Riyadh, SAU; 2 Medicine, King Saud University, Riyadh, SAU

**Keywords:** cardiac surgery, cardiac trauma management, chest trauma, emergency cardiac surgery, penetrating cardiac injuries, penetrating chest trauma, trajectory of injury, traumatic cardiac injury

## Abstract

A 37-year-old man presented with a gunshot wound to the left chest. Initial evaluation revealed no exit wound, and a left anterolateral thoracotomy was performed, revealing a right ventricular injury. A transesophageal echocardiogram (TEE) identified the bullet lodged in the right ventricle near the septal leaflet of the tricuspid valve. Given the bullet's trajectory, a median sternotomy was performed for the removal of the bullet and the repair of the ventricular septal defect. The patient made a full recovery postoperatively. This case highlights the critical role of anticipating and understanding bullet trajectory in guiding surgical decision-making and optimizing patient outcomes.

## Introduction

A stab wound or bullet to the chest can reach the heart in seconds, creating a life-threatening emergency that demands split-second decisions. Penetrating cardiac injury, though uncommon, represents one of the deadliest forms of thoracic trauma, with many patients dying before reaching the hospital and others deteriorating rapidly in the emergency department [1]. The nature of cardiac injury depends on the weapon involved. Stab wounds typically create a direct puncture track, while gunshot wounds transfer kinetic energy that can damage tissue beyond the projectile's path. Once the heart is penetrated, two life-threatening processes begin immediately: severe bleeding leading to inadequate blood volume (hypovolemic shock) and cardiac tamponade, a condition where blood fills the pericardial sac surrounding the heart, compressing it and preventing normal filling. Together, these create obstructive shock that can progress to death within minutes [2].

Diagnosis begins with understanding the mechanism of injury and recognizing signs of deterioration: unstable blood pressure, distended neck veins from increased venous pressure, muffled heart sounds, and evidence of blood in the chest cavity. Modern diagnosis relies heavily on point-of-care ultrasonography, including extended focused assessment with sonography for trauma (eFAST) and focused transthoracic echocardiography, which can rapidly detect pericardial fluid. When patients are hemodynamically stable, contrast-enhanced computed tomography (CT) can delineate the exact path of the weapon and identify concomitant thoracic injuries.

Treatment options span a spectrum based on injury severity and patient stability. The most critical patients require immediate resuscitative thoracotomy and pericardiotomy in the emergency department to relieve cardiac tamponade and control hemorrhage. Others proceed to the operating room for median sternotomy, surgical division of the breastbone, followed by pledgeted repair of cardiac lacerations using reinforced sutures. Complex cases involving coronary arteries or heart valves may necessitate cardiopulmonary bypass, where a heart-lung machine temporarily assumes cardiac function during repair [3].

Clinical decision-making is complicated by multiple factors: occult chamber perforations that may not be immediately apparent, coronary vessel injury, retained foreign bodies, coagulopathy, and narrow windows for safe imaging in unstable patients. Beyond individual patient factors, system barriers include delayed transport from the injury scene to the hospital, limited access to round-the-clock echocardiography or cardiac surgery, and the challenge of rapidly coordinating multidisciplinary teams comprising trauma surgeons, anesthesiologists, and perfusion specialists in time-critical settings. Understanding the exact trajectory of the penetrating object through the chest is fundamental to surgical planning because it predicts which cardiac chambers are injured, determines the optimal surgical incision, and helps anticipate complications such as life-threatening bleeding or disruption of the heart's electrical conduction system.

We present a case in which meticulous trajectory assessment using transthoracic echocardiography and CT guided a targeted surgical approach and expedited hemorrhage control, underscoring how trajectory awareness can optimize outcomes in high-risk penetrating cardiac trauma [4].

## Case presentation

**Figure 1:**
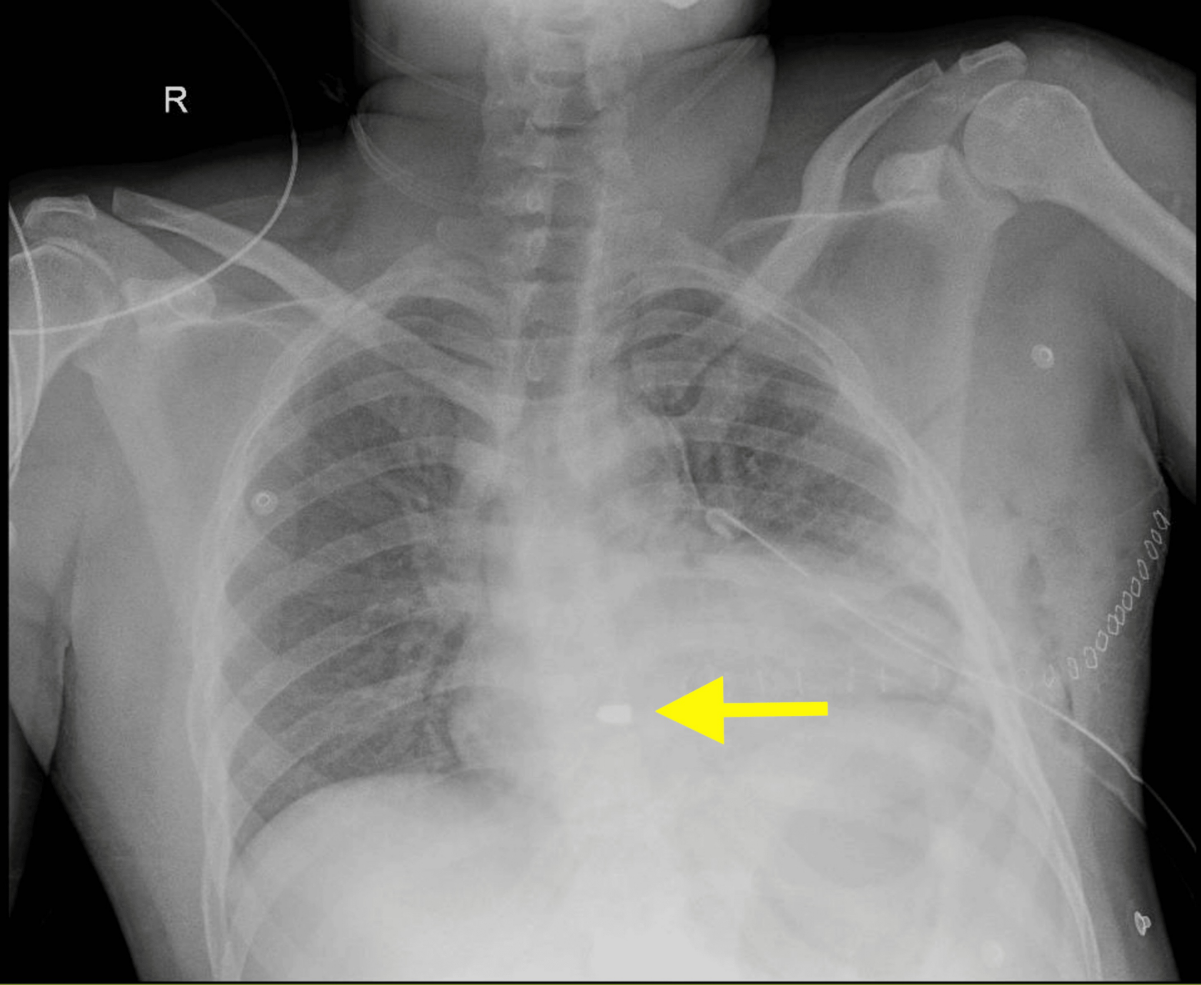
Chest X-ray illustrating the location of the bullet

A 37-year-old male patient with no known pre-existing medical conditions presented to the emergency department (ED) with a history of a gunshot wound to the chest. On examination, a 2 cm wound was identified in the left fifth intercostal space, near the left midclavicular line. The patient was normotensive, maintaining room air saturation, with a Glasgow Coma Scale (GCS) of 15/15. Cardiovascular and respiratory examinations were unremarkable. A chest X-ray revealed a bullet lodged, most likely, in the right ventricle (Figure 1).

However, an eFAST showed a large pericardial effusion at the time of presentation. Subsequently, the patient became hypotensive, leading to his transfer to the operating room (OR) for emergency thoracotomy. In the OR, a total of 200 mL of pericardial blood was evacuated, and a 2 cm right ventricular defect was repaired.

**Figure 2:**
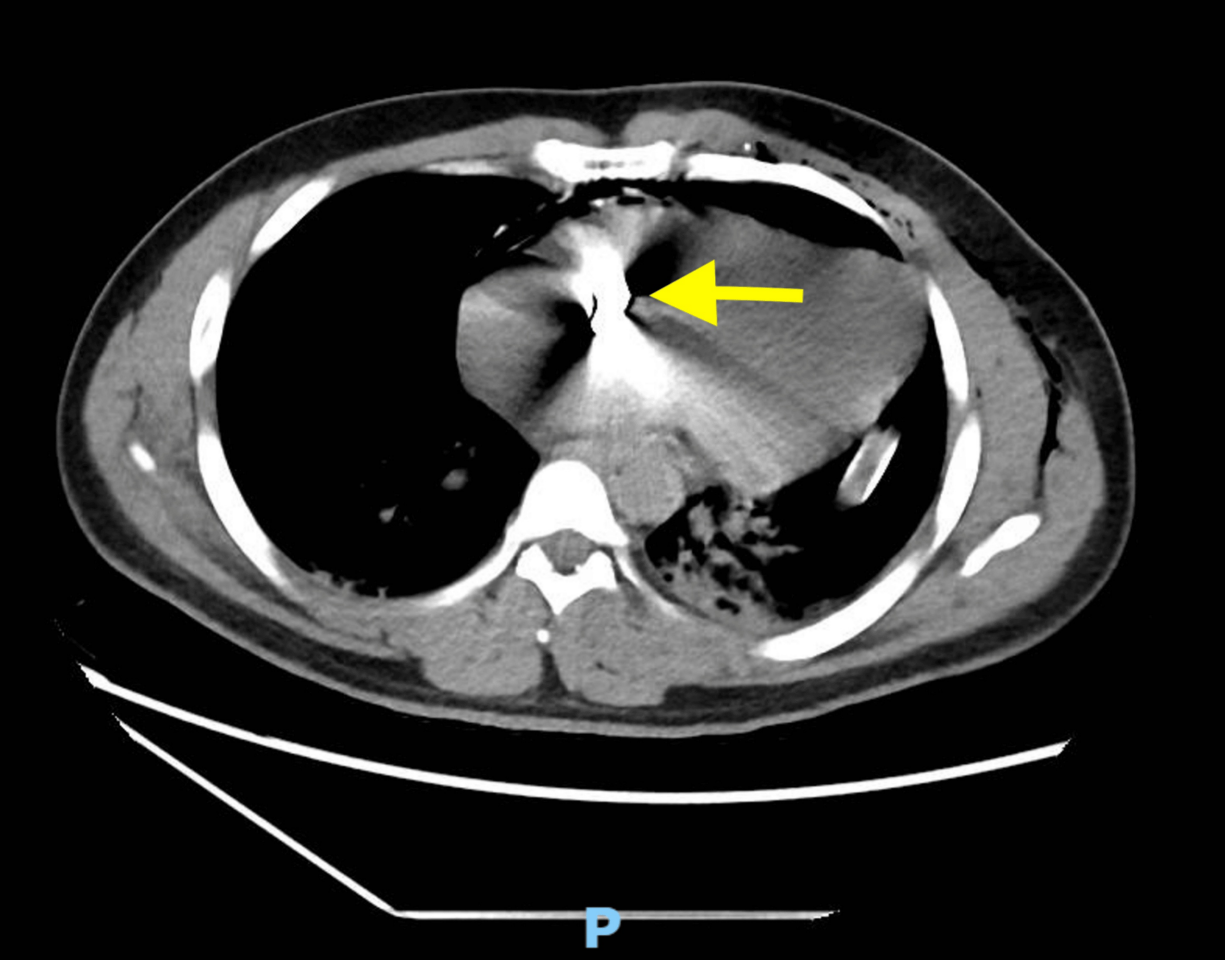
A chest CT illustrating the location of the bullet

Postoperatively, he was transferred to the intensive care unit (ICU) for further evaluation. The chest CT displayed a high likelihood of the bullet's position between the right atrium near the right atrioventricular junction and the anterior inferior aspect of the ascending aorta (Figure 2).

**Figure 3:**
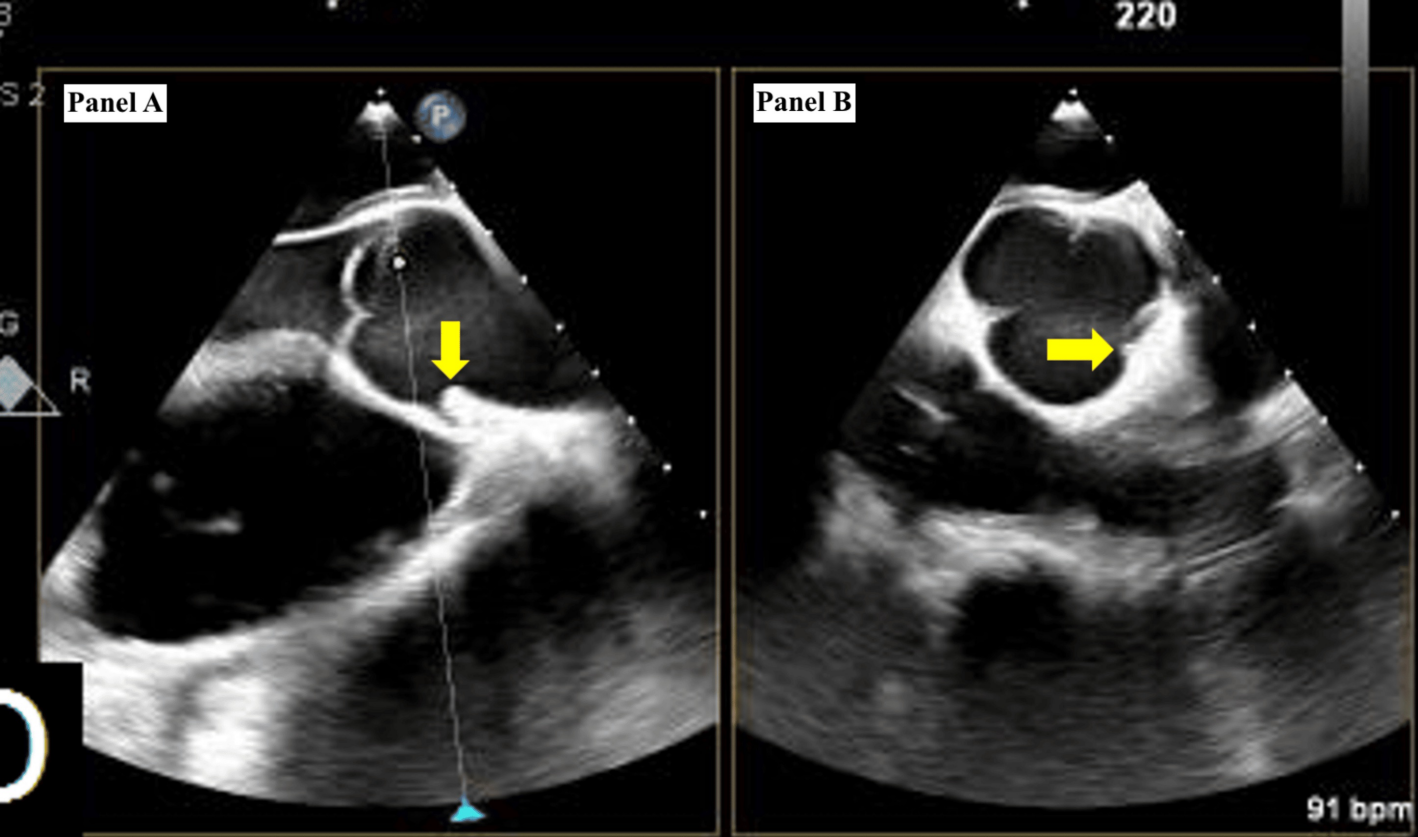
A transesophageal echocardiogram (TEE) illustrating the location of the bullet near the tricuspid valve

A transesophageal echocardiogram (TEE) was performed and showed an echogenic mass seen in the right atrium, near the annulus of the tricuspid valve (Figure 3).

**Figure 4:**
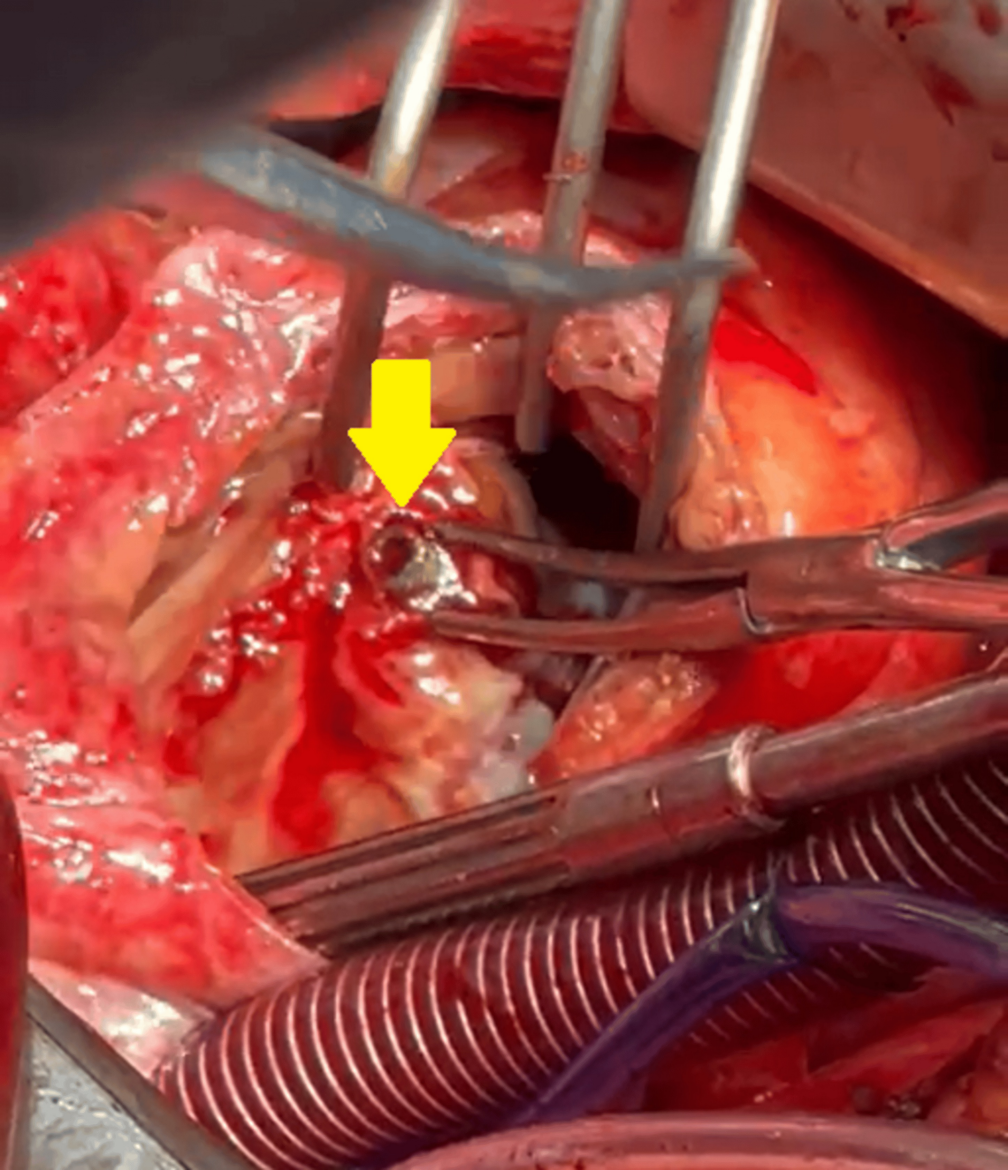
An intraoperative image showing the extraction of the bullet

After three days in the ICU to ensure complete stabilization of his condition, the patient underwent bullet removal performed by the cardiac surgery team via median sternotomy. Cardiopulmonary bypass was performed by bicannulation of the superior vena cava and inferior vena cava, as there was no need for peripheral cannulation. The patient was cooled to 32°C, and cold blood cardioplegia was given. A right atriotomy incision was made to access the bullet located near the septal leaflet of the tricuspid valve and conduction system. The bullet was embedded at the base of the septal leaflet of the tricuspid valve, which has to be detached from its insertion and reattached afterwards using continuous 5/0 Prolene suture (Ethicon, Inc., Somerville, New Jersey, USA; Figure 4).

**Figure 5:**
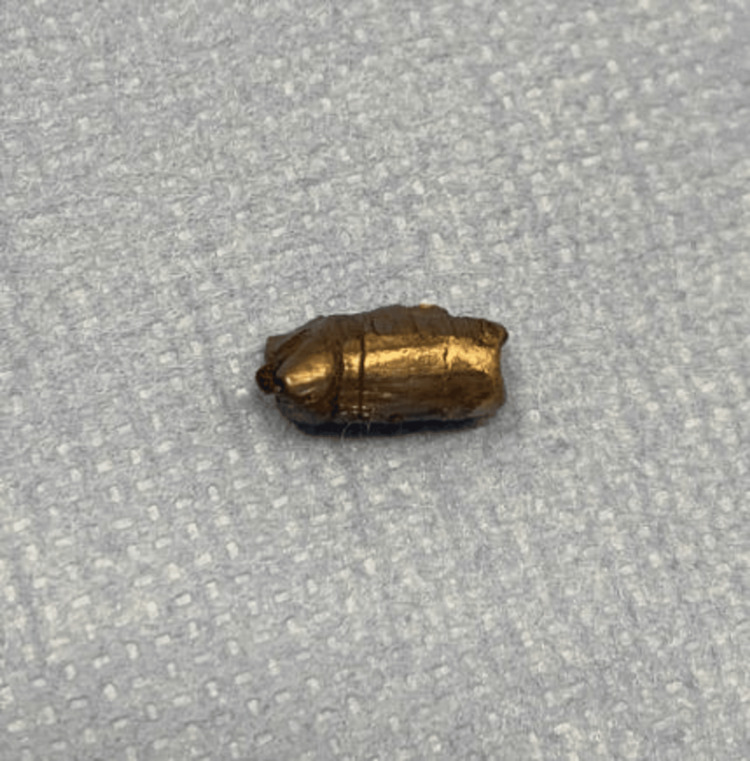
Image of the extracted bullet

The bullet was successfully visualized and removed (Figure 5). 

The resulting ventricular septal defect was repaired using a bovine pericardial patch. The total bypass time was 71 minutes, including 53 minutes of cross-clamp time. An intraoperative TEE revealed a competent tricuspid valve, which warranted no further maneuvers. The surgery was uncomplicated, and the patient was discharged on the fifth postoperative day. He was later seen in the clinic for follow-up. During follow-up, the patient had normal cardiac function, clean wounds, and no signs of deterioration.

## Discussion

Penetrating cardiac injuries represent approximately 10% of all thoracic trauma cases, with incidence varying significantly by geographic region and socioeconomic factors. Urban trauma centers in high-violence areas report penetrating cardiac injury rates as high as 15-20 per 100,000 population annually, predominantly affecting males aged 20-40 years. Overall mortality for penetrating cardiac trauma ranges from 50% to 80%, with prehospital mortality accounting for the majority of deaths. However, survival varies dramatically based on the mechanism of injury, anatomical location, and time to definitive intervention. Stab wounds carry a 70% to 80% survival rate when patients reach the hospital alive, compared to only 20% to 40% for gunshot wounds, reflecting the greater kinetic energy transfer and tissue destruction associated with ballistic trauma [5].

The mechanism of cardiac penetration follows predictable anatomical patterns based on the site of entry. Anterior chest wounds typically affect the right ventricle (the most anterior chamber), followed by the left ventricle. Left lateral entry wounds predominantly injure the left ventricle and circumflex coronary artery, while posterior wounds may involve the atria, posterior descending artery, and esophagus. Subxiphoid wounds carry a particular risk for right ventricular and hepatic injury. Shotgun injuries at close range produce devastating wounds, with multiple pellets creating a constellation of cardiac perforations [6].

Projectile composition and design significantly influence injury patterns and outcomes. Full metal jacket bullets tend to pass through the heart, creating entrance and exit wounds, while hollow-point and soft-nose projectiles expand upon impact, transferring maximum energy and causing catastrophic tissue destruction with lower exit probabilities. Retained intracardiac foreign bodies occur in approximately 30% of ballistic cardiac injuries, with location determining clinical urgency; projectiles in ventricular cavities risk systemic embolization, those near valvular structures threaten mechanical dysfunction, and fragments in the conduction system may cause dysrhythmias. Studies demonstrate that survival with retained intracardiac projectiles approaches 90% when promptly recognized and surgically addressed, compared to 40% to 50% when diagnosis is delayed beyond 24 hours [7].

Management strategies for penetrating cardiac trauma have evolved considerably, with contemporary approaches emphasizing rapid decision-making based on hemodynamic status and injury pattern. Unstable patients with signs of tamponade require immediate resuscitative thoracotomy, typically via a left anterolateral incision, which allows rapid pericardiotomy, hemorrhage control with digital pressure or simple suturing, and internal cardiac massage if necessary. The resuscitative endovascular balloon occlusion of the aorta (REBOA) technique has emerged as a temporizing measure in exsanguinating patients, with zone I occlusion providing afterload enhancement and reduced subdiaphragmatic hemorrhage during transport to the operating room [8].

For hemodynamically stable patients, cannulation strategies must be carefully planned: peripheral cannulation (femoral vessels) is preferred when median sternotomy is anticipated, while central cannulation (aortic and atrial) provides superior flow rates for complex repairs requiring cardiopulmonary bypass. Emergency cardiopulmonary bypass is indicated in approximately 15% to 20% of operative cases, particularly for injuries involving coronary arteries, valvular structures, or complex septal defects [9].

The timing and technique of projectile removal remain controversial and require individualized assessment. Immediate extraction is mandatory for projectiles causing tamponade, valvular dysfunction, or systemic embolization risk. Conversely, deeply embedded fragments in the myocardium or septum without mechanical complications may be observed, as extraction attempts risk precipitating hemorrhage or creating larger defects requiring complex reconstruction. Small caliber bullets in stable locations have been successfully managed non-operatively in 30 to 40% of cases in contemporary series, with serial echocardiography monitoring for delayed complications including vegetation formation, progressive valvular regurgitation, or late embolization. When extraction is undertaken, the surgical approach depends on projectile location: right atrial fragments are accessed via median sternotomy with bicaval cannulation, left ventricular projectiles may require left thoracotomy for optimal exposure, and septal foreign bodies often necessitate cardiopulmonary bypass with cardioplegic arrest to prevent massive hemorrhage during retrieval [10].

In the present case, the initial diagnostic workup utilizing chest radiography and eFAST rapidly identified pericardial effusion and hemothorax, prompting immediate surgical exploration. The reported 27% survival rate for this injury severity, a through-and-through right ventricular wound with a retained intracardiac projectile, reflects the critical nature of such injuries and underscores the importance of aggressive intervention [5]. Emergency thoracotomy enabled pericardial decompression and primary repair of the ventricular defect, preventing further hemodynamic deterioration. The subsequent decision to pursue computed tomography and transesophageal echocardiography, once the patient was stabilized, provided crucial trajectory information and identified the bullet's precarious location near the tricuspid valve [11]. The multidisciplinary team's decision to proceed with median sternotomy for definitive projectile extraction was justified by the substantial risks of non-intervention: septic endocarditis (reported in 20% to 30% of retained projectiles), systemic embolization (15% to 25% risk for right-sided projectiles reaching pulmonary circulation), progressive valvular dysfunction, and conduction abnormalities [7].

Outcomes for penetrating cardiac trauma depend on multiple prognostic factors beyond the mechanism of injury. The Cardiac Injury Scoring System stratifies patients from grade I (tangential wounds not requiring repair) to grade V (avulsion of cardiac chambers), with grade III injuries (through-and-through chamber wounds) carrying 40% to 60% mortality despite optimal care. Additional negative prognostic indicators include cardiac arrest at presentation (5% to 10% survival), injuries to multiple cardiac chambers (20% to 30% survival), and associated great vessel injuries (15% to 25% survival). Conversely, positive predictors include isolated right ventricular injury, preserved mentation upon emergency department arrival, and door-to-operative-intervention time under 30 minutes [12].

Long-term complications in survivors include post-traumatic ventricular septal defects (1% to 5% incidence), valvular regurgitation (3% to 4%), pericardial effusions (8% to 9%), and coronary artery pseudoaneurysms (<2%), with rare cases of constrictive pericarditis, necessitating extended surveillance with serial echocardiography [13]. This case exemplifies the critical importance of systematic trajectory assessment, coordinated multidisciplinary care, and aggressive surgical management in optimizing outcomes for penetrating cardiac trauma. Future research should focus on refining prehospital triage algorithms to identify salvageable patients, developing predictive models to guide decision-making regarding projectile extraction versus observation, and investigating adjunctive therapies such as hemostatic agents and damage control resuscitation protocols. Additionally, population-level interventions addressing firearm violence and trauma prevention remain essential to reducing the burden of penetrating cardiac injuries.

## Conclusions

Trajectory-directed planning should anchor operative strategy in penetrating cardiac trauma. This case demonstrates a staged approach: emergency thoracotomy for pericardial decompression and right ventricular repair, followed by CT and TEE localization, then definitive median sternotomy with cardiopulmonary bypass for projectile extraction and ventricular septal defect repair. This physiology-first sequence prevented valvular injury, conduction abnormalities, and embolic complications, yielding discharge on postoperative day five with normal cardiac function at follow-up. The case supports a practical algorithm: stabilize immediately, delineate trajectory with multimodal imaging, then tailor surgical approach to optimize survival and functional outcomes.
